# The multifaceted roles of B lymphocytes in metabolic dysfunction–associated steatotic liver disease

**DOI:** 10.3389/fimmu.2024.1447391

**Published:** 2024-09-20

**Authors:** Huige Li, Ning Xia

**Affiliations:** Department of Pharmacology, University Medical Center, Johannes Gutenberg University, Mainz, Germany

**Keywords:** metabolic dysfunction-associated steatohepatitis, metabolic dysfunction-associated steatotic liver disease, B lymphocytes, NAFLD, NASH

## Abstract

Recent evidence suggests that adaptive immune cells are important contributors to metabolic dysfunction–associated steatotic liver disease (MASLD, formerly non-alcoholic fatty liver disease, NAFLD). In liver biopsies from MASLD patients, the accumulation of intrahepatic B cells is positively correlated with the MASLD activity score. Hepatic B-cell infiltration is observed in experimental models of metabolic dysfunction-associated steatohepatitis (MASH, formerly non-alcoholic steatohepatitis, NASH). Intrahepatic B2 cells have been shown to contribute to MASLD/MASH by activating T cells, macrophages and hepatic stellate cells, and by producing pathogenic IgG antibodies. In mice fed a MASH diet, selective depletion of B2 cells reduces steatohepatitis and fibrosis. Intestinal B cells are metabolically activated in MASH and promote T-cell activation independently of TCR signaling. In addition, B cells have been shown to contribute to liver fibrosis by activating monocyte-derived macrophages through the secretion of IgA immunoglobulins. Furthermore, our recent study indicates that certain B cell subsets, very likely regulatory B cells, may play a protective role in MASLD. This review summarizes the molecular mechanisms of B cell functions and discusses future research directions on the different roles of B cells in MASLD and MASH.

## 1 Introduction

With worldwide increasing prevalence of obesity, metabolic dysfunction–associated steatotic liver disease (MASLD, formerly non-alcoholic fatty liver disease, NAFLD) has become a global health problem affecting up to one-third of the world’s adult populations (1–3). Metabolic dysfunction-associated steatohepatitis (MASH, formerly non-alcoholic steatohepatitis, NASH) is the aggressive form of MASLD and characterized by steatosis, inflammation, hepatocellular injury, and fibrosis. MASH can progress to cirrhosis and/or hepatocellular carcinoma and has become the leading cause of liver-related morbidity and mortality worldwide, and a primary indication for liver transplantation (4, 5).

As the population ages and the duration of exposure to the disease increases, the majority of models predict a significant rise in the prevalence of MASLD-related cirrhosis and end-stage liver disease (6). MASLD is the most rapidly growing contributor to liver-related mortality and morbidity (1). Thus, there is an urgent need to develop new, effective pharmacological therapies for MASH/MASLD and its complications.

Inflammation is key in the progression from hepatic lipid accumulation to the more severe MASH (7). In fact, the onset of the inflammatory response is an early event that precedes the progression toward MASH (8). Importantly, MASLD is a progressive disease. Although immune mechanisms contribute to all stages of MASLD, the immune response may differ in the initial phase of lipid accumulation than in the stage of MASH. For instance, CD8^+^ T cells promote metabolic dysregulation in the early stage of steatosis whereas they cause hepatocyte death at later stages (9, 10).

Immune responses are a major part of the inflammatory process in MASH (9). Both innate and adaptive immune cells are implicated in the pathogenesis of MASLD (11). The innate immune response in MASH has been the focus of research and it is well established that innate immune cells make a major contribution to inflammation in MASH (12–14). In addition to Kupffer cells, further innate immune cells involved in MASLD include neutrophils, monocytes/macrophages, dendritic cells, natural killer T cells, mucosal-associated invariant T cells, and innate lymphoid cells (ILCs) (11, 13). Especially monocyte-derived macrophages (MoMFs) can directly activate hepatic stellate cells (HSC) and are of particular importance in the context of liver injury and fibrosis in MASH (15, 16). ILCs represent the most recently discovered innate immune cell family and consists of 5 subsets: natural killer (NK) cells, type 1, 2 and 3 ILCs and lymphoid tissue inducer cells (17, 18). Liver type ILC1s are expanded in cirrhotic livers of patients (19) and their roles in liver disease are subject of current research (11, 17). Nevertheless, growing evidence indicates that adaptive immune cells also play an essential role in MASLD (11, 13, 14, 20). The present review article focuses on the role of B lymphocytes.

## 2 General mechanisms of B cell development, activation and function

### 2.1 B cell development

During early fetal development, human B-lineage cells are found in various fetal tissues. However, starting from midgestation, bone marrow becomes the sole site for B lymphopoiesis (21). In the bone marrow, common lymphoid progenitor (CLP) cells can differentiate into early-pro-B cells, and then into pro-B cells. A process of gene recombination occurs through the rearrangement of genes coding for segments V (*Variable*), D (*Diversity*), and J (*Joining*) of the heavy chain (H chain) together with that of the genes for segments V and J of the light chain (L chain) of the membrane-bound immunoglobulin (mIg). The early-pro-B cells are typically characterized by DJ_H_ rearrangement initiation, and pro-B cells by VDJ_H_ rearrangement. A proper VDJ_H_ rearrangement is necessary for pro-B cells to differentiate into pre-B cells. Pro-B cells without functional VDJ_H_ rearrangement will cease to develop, either undergoing apoptosis or being phagocytized by bone marrow macrophages (21). Pro-B cells express no surface immunoglobulin molecules, whereas pre-B cells express the so-called surrogate light chains paired with the µH chain forming the pre-BCR on the membrane (22). The pre-BCR is essential for pre-B cell proliferation and further differentiation into immature B cells expressing IgM as the complete BCR (22).

Bone marrow-derived immature B cells can mature in bone marrow, or migrate from the bone marrow to the spleen, and differentiate, through transitional stages of maturation (T1 and T2 B cells), into either follicular B cells or marginal zone B cells expressing IgM and IgD. Now they are considered mature B cells or naïve B cells (23).

### 2.2 B cell activation

Naïve B cells get activated in secondary lymphoid organs (spleen, lymph nodes, Peyer’s patches, tonsils, and mucosal tissue) by antigen sensing via the B cell receptor (BCR) (24). In addition to BCR, complex antigens also engage co-receptors on B cells, such as toll-like receptors (TLRs) or complement receptors (CR2), leading to potentiation of the BCR signal. If the signaling via co-receptors is strong, B cells start to proliferate without the need for T cell help, resulting in a T cell-independent type 1 response. Alternatively, B cells can be activated via a T cell-independent type 2 response, if the BCR stimulation by highly multivalent antigens alone is strong enough (24). These T-independent processes can be enhanced by cytokines like BAFF and APRIL (24).

T cell-dependent antigens such as foreign proteins usually activate B cells with the help of T cells (24). After binding to BCR, the antigen is taken up by B cells, degraded, and presented on the cell membrane as peptide pieces in complex with MHC-II (25). The complex is recognized by T helper cells, and bound by T cell receptor (TCR), leading to expression CD40 ligand (CD40L) on the T cell surface. CD40L binds CD40 on B cell surface providing a co-stimulation for B cell activation. In addition, T helper cells secrete cytokines, such as IL-4 and IL-21, promoting B cell proliferation, class switch recombination, and differentiation into antibody-producing cells or germinal center B cells (24).

### 2.3 B cell function

Class switch recombination allows activated B cells to change the isotype of their BCR from IgM and IgD to IgG, IgA, or IgE, leading to the generation of antibody-secreting B cells (plasmablasts and plasma cells) (22, 24, 25). Antibodies can mediate their effector functions by binding to Fc receptors on target cells or by activating the complement system (24). In addition to antibody production, B cells also function via antigen presentation, cell-cell interaction, and cytokine secretion (22).

## 3 Genetic mouse models of B cell-deficiency

To study the role of B cells, both therapeutic and genetic approaches are available. Using an anti-IgM depleting antibody, 90-95% mature B cells can be deleted (26). However, plasma cells are not depleted by this antibody. The approach applying anti-CD20 antibodies is more efficient, resulting in 95% B cell depletion (27). Anti-CD20 antibodies don’t deplete plasma cells either, as CD20 is not expressed on plasma cells (28). In contrast, complete B cell-deficiency can be reached in genetically modified mice. Currently, there are several B cell-deficient mouse lines used in research (**Table 1**).

**Table 1:** Selected findings in genetically modified mice.

Genotype	B cells	Secreted antibodies	Diet	Liver steatosis	ALT, AST	Mean Score	MASH	Liver fibrosis	Ref.
JH^-/-^	No	No	CD-HFD	No	Normal	1.1	No	No	(16)
IgMi	Yes	No	CD-HFD	No	Normal	1.6	No	No	(16)
IgA^-/-^	Yes	lacking IgA	CD-HFD	Yes	Increased	n.d.	Yes	No	(16)
µMT	Only gut IgA^+^ B cells	only IgA	CD-HFD	≈ WT	≈ WT	4.8	≈ WT	No	(16)
µMT	Only gut IgA^+^ B cells	only IgA	HFHC	< WT	< WT	3.5	< WT	< WT	(40)
JHT	No	No	HFD	improved	Normal	n.d.	improved	improved	(36)
IgMi	Yes	No	HFD	No	Normal	n.d.	No	No	(36)

ALT, alanine transaminase; AST, aspartate transaminase; CD-HFD, choline-deficient high-fat diet; HFD, high-fat diet; HFHC, high-fat high-carbohydrate diet; mean score, average MASLD activity score; n.d., no data; WT, wildtype mice.

### 3.1 The µMT mice

The µMT mice were generated by disrupting one of the membrane exons of the gene encoding the µ-chain constant region (29). µ-chain is the heavy chain of IgM antibodies and the heavy chain of the pre-BCR expressed on the membrane of developing B cells. This pre-BCR is essential for pre-B cell differentiation. Homozygous mice with µ-chain gene disruption lack immature and mature B cells. The mutants have large pre-B cells, but not small pre-B cells, indicating that B cell development is arrested at the stage of pre-B cell maturation (29). Although BALB/c mice with the μMT mutation are “leaky” and generate small numbers of mature B cells, this phenomenon is not observed in mutant mice on the C57BL/6 background (28, 30). Interestingly, µMT mice develop IgA^+^ B cells in the absence of IgM, independent of genetic background (31). IgA^+^ cells are found in the spleen and ileum, with no other B cell populations in µMT spleen or bone marrow. It is known that IgD can substitute for IgM in early phase of B cell development. However, the generation of IgA^+^ B cells in µMT mice is independent of IgM or IgD (31). Rather, it requires the specialized nature of the gut, where extrafollicular B cells are the major producers of IgA (32). It is proposed that some of the early B cell precursors leave the bone marrow and receive their switching signals (TGF-β and gut commensal bacterial antigens) in gut lymphoid tissues such as Peyer’s patches, and thus bypassing the requirement for IgM or IgD expression (31, 32).

### 3.2 The JHT mice

The JHT mice were generated by deleting the loci for the heavy chain joining region (J_H_) and the E_µ_ intron enhancer (J_H_–E_µ_) of the IgH gene using a Cre-loxP recombination system (33). The rearrangement of the V_H_-D_H_-J_H_ rearrangement requires the presence of both J_H_ and E_µ_ (34). As a result, the formation of heavy chain V region genes through somatic V_H_-D_H_-J_H_ rearrangement is impossible in the JHT mice. The mutant mice cannot make functional immunoglobulin molecules and the animals are completely avoid of mature B cells in all tested genetic background (28, 33). Without membrane-bound immunoglobulin molecules, the development of B cells is blocked at the pre-B cell stage in the homozygous mice, because pre-BCR is essential for B cell development (29, 33).

### 3.3 The JH^-/-^ mice

The JH^-/-^ mice were generated at a different laboratory (34). In these mutant mice, the J_H_ gene segments (but not the E_µ_ intron enhancer) in the IgH locus are deleted. As a result, B cell development is blocked at the precursor stage, with increased number of CD43^+^ pro-B and pre-B cells in the bone marrow, but lacking the more mature CD43^-^ pro-B cells. The homozygous mutant mice show a complete absence of mature B cells in the bone marrow, spleen or peripheral blood (34). The IgH gene assembly is initiated by joining of a D_H_ to a J_H_ gene segment, followed by rearrangement of a V_H_ to the DJ_H_ complex (34). In the homozygous mutant mice with J_H_ gene segment deletion, however, the V_H_ to D-J_H_ joining doesn’t occur, indicating that D-J_H_ rearrangement is required for V_H_ to D-J_H_ joining, and J_H_ gene segment essential for IgH gene assembly (34). As mentioned above, pro-B cells cannot develop further without functional VDJ_H_ rearrangement (21).

### 3.4 The IgMi mice

The IgMi mice were generated by deleting the polyA responsible for the secreted form of antibodies, but allowing the expression of immunoglobulin molecules on B cell surface (i.e. BCR). Two polyA sites are present in the constant region coding for the heavy chain of IgM. The first polyA site is essential for the maturation of the RNA for the secreted form of the IgM heavy chain, whereas the second polyA site is responsible for the maturation of the RNA for membrane-form of the IgM heavy chain. In the IgMi mice, only the first polyA site is deleted while the second polyA site is kept intact. As a result, these mice express IgM as BCR on the B cell surface enabling B cell development. However, class switch recombination is impossible in these B cells and the cells cannot differentiate into antibody-producing cells. The IgMi mice represent a unique tool to distinguish the B cell roles as antibody producer from other B cell functions (28, 35). The IgMi mice don’t secrete antibodies, but they have normal B cell number of B cells (36, 37), produce cytokines and their adaptive immune responses remain largely intact (16).

## 4 Intrahepatic B2 cells in MASLD and MASH

Lymphocyte infiltration is one of the histological features of MASLD. In murine models of MASLD, B cells are among the first to accumulate in response to high-fat diet (HFD) feeding (38, 39). In liver biopsies from MASLD patients, intrahepatic B cell accumulation positively correlates with the MASLD activity score (40). In patients with MASH, focal aggregates of B cells and T cells are formed, which resemble ectopic lymphoid structures (13, 41). These aggregates’ size and frequency show a direct association with lobular inflammation and fibrosis scores (41). Hepatic infiltration by B cells is also evident in various experimental models of MASH (41, 42).

B cells are among the most frequent immune cell types in the liver and comprise as much as 20% - 30% of all CD45^+^ immune cells in the mouse liver under normal chow conditions (40), although much fewer B cells are found in human liver (12, 43). In response to high-fat high-carbohydrate (HFHC) diet feeding, the percentage of B cells among the immune cells remains unchanged, but the total number of immune cells increases significantly. As a result, the absolute number of B cells in the mouse liver almost doubles after 15 weeks of HFHC (40). The majority of the intrahepatic B cells are IgM^+^ IgD^+^ B2 cells (CD3^−^ NK1.1^−^ CD19^+^ CD23^+^ CD5^−^ B220^hi^) producing TNF-α and IL-6, and the number of B2 cells is increased in MASH mice (40). Consistently, intrahepatic B cell accumulation is also evident in liver biopsies from MASLD patients (40).

### 4.1 Activation

T cell-independent mechanism seems to play an important role in the activation of intrahepatic B cells during MASLD/MASH. It is likely that gut-derived microbial antigens or gut microbiota metabolites, such as LPS, activate B cell by binding to BCR and TRL4 simultaneously (40). TLR4 activation in MASH is not only limited to Kupffer cells or hepatocytes (44), but B cells can respond to pathogen-derived ligands through TLR4 signaling as well (45). TRL4 serves as a co-receptor for B cell activation through the MYD88 pathway (40). Consistently, B cell–specific depletion of MyD88 ameliorates MASH progression (40). T-independent B cell-activation can be enhanced by cytokines like BAFF and APRIL (24). Indeed, BAFF levels are increased in MASH patients (46). B cells from HFHC-fed mice show MyD88-dependent upregulation of MHC I, MHC II and CD86, indicating activation and increased capability of antigen presentation (40).

**Figure 1:**
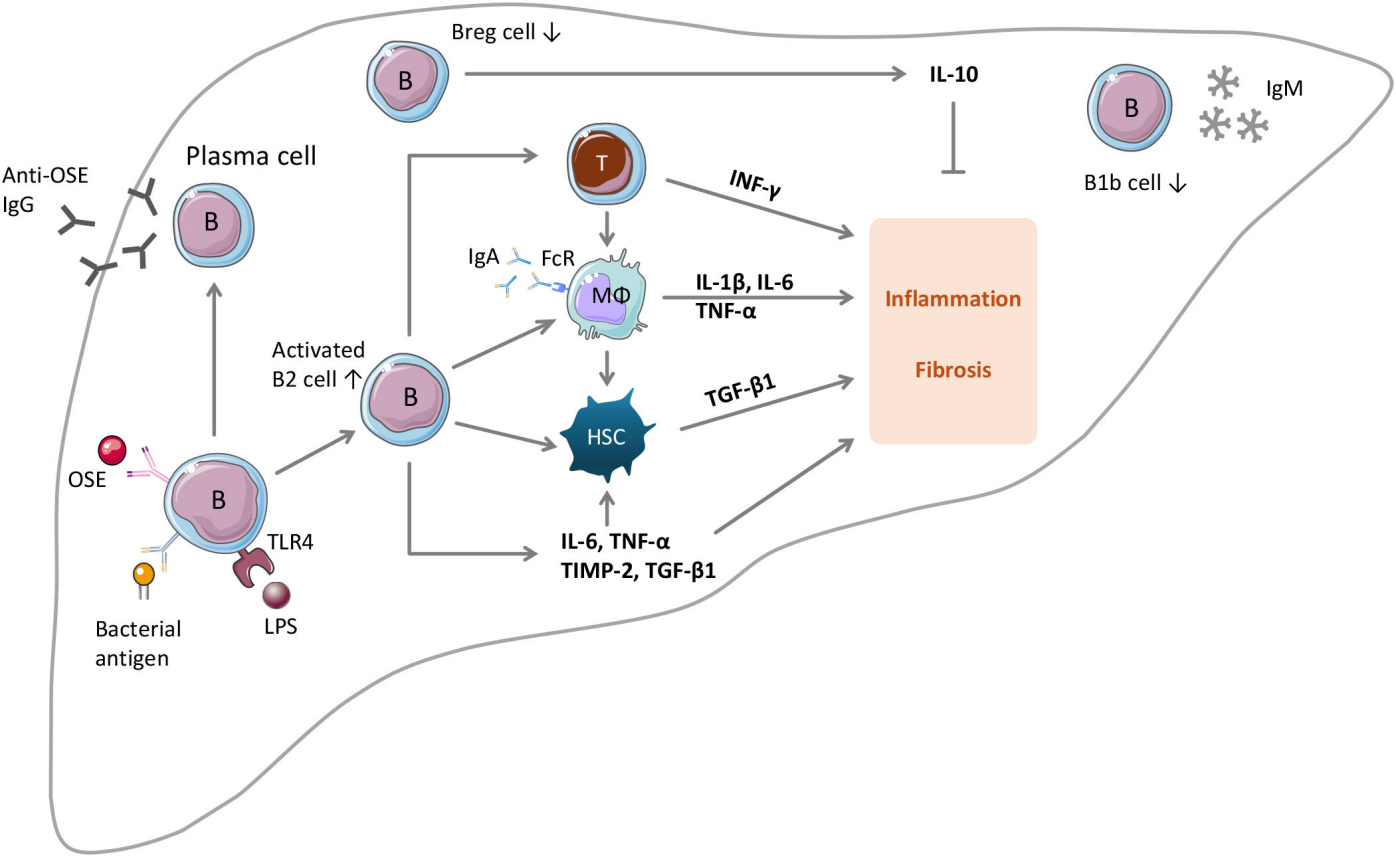
The roles of B lymphocytes in MASLD and MASH. Upon stimulation by OSE, B cells can develop into IgG-producing plasma cells. Intrahepatic B cells can be activated by gut bacterial antigens or by gut microbiota metabolites such as LPS. Under the conditions of MASLD and MASH, the number of B2 cells in the liver increases, whereas the numbers of B1b cells and regulatory B cells (Breg) decrease. Activated B2 cells promote MASLD and MASH through multiple mechanisms: (i) activation of CD4 and CD8 T cells by antigen presentation and by secreting IL-6 and TNFα; (ii) activation of HSC by releasing TNFα; (iii) activation of macrophages in an IgA- and Fc receptor-dependent manner; (iv) by producing pro-inflammatory and profibrotic factors; (v) by releasing anti-OSE IgG antibodies. IL-10 produced by Breg cells has protective effects. The role of B1b cells is unknown.

The effects of B cells can be mediated by interaction with other cell types, by secretion of cytokines, or by antibody production. Indeed, B cells contribute to MASLD and MASH through multiple mechanisms (**Figure 1**).

### 4.2 Interaction with T cells

The principal T lymphocytes participating in adaptive immunity comprise CD4^+^ T cells, CD8^+^ T cells, and γδ T cells. CD4^+^ T cells can be further classified into subsets such as T helper (Th) 1, Th2, Th17, regulatory T (Treg) cells. Th1 and Th17 subsets promote MASLD progression by producing IFN-γ and IL-17, whereas the roles of Th2 and Treg are incompletely understood (11). Blocking CD4^+^ T cell recruitment to the liver protects mice from MASLD, supporting the crucial role of these cells (47). In contrast, the role of CD8^+^ T cells in MASLD pathogenesis varies depending on the context (11). Under the condition of MASH, CD8^+^ T cells can become auto-aggressive, killing hepatocytes in a MHC-I-independent manner (48). On the other hand, tissue-resident memory CD8^+^ T cells have been shown to promote the resolution of hepatic inflammation and fibrosis by causing apoptosis of HSCs (49).

The effects of B lymphocytes in MASLD are partly mediated by interaction with T cells.

In patients with MASH, liver biopsies demonstrate the presence of B-cell aggregates within T-cell-rich regions, indicating a potential interaction between these cell types (41). In steatosis (38) and MASH (40), intrahepatic B cells secrete elevated levels of IL-6 and TNFα, which promote the activation of CD4 cells and their differentiation into Th1 cells.

In mouse models of MASH, B-cell activation parallels the onset of steatohepatitis (41). Moreover, the B2 cell response precedes T-cell activation and is accompanied by upregulation of the hepatic expression of BAFF, a cytokine driving B cell survival and maturation. The circulating levels of BAFF are increased in MASH patients, and correlates with the severity of steatohepatitis and fibrosis (46). BAFF-deficient mice are partially protected from MASH (50). Treatment with the BAFF-neutralizing monoclonal antibody Sandy-2 prevents hepatic B2 cell responses and ameliorates established MASH in mice (41). Selective depletion of B2 cells in mice reduces steatohepatitis and fibrosis in mice fed MASH-diets (41).

Theoretically, it requires three signals for B cells to activate T cells: (i) antigen presentation to T cell receptor thereby promoting T cell proliferation and differentiation; (ii) co-stimulatory signals stabilizing the immunological synapse between B cells and T cells, and (iii) cytokine released by B cells to shape the polarization of T cells towards an effector phenotype (51). Indeed, the activation of B cells in MASH is associated with an upregulation of MHC-I and MHC-II indicating enhanced antigen-presenting ability (40, 41). The protein antigens are likely derived from gut bacteria, which are processed by B cells as professional antigen presenting cells. The resulting peptides are presented to T cells by forming complexes with MHC molecules. In addition, the activated B cells show also enhanced expression of CD86 (40, 41). CD86 binding with CD28 on T cells represents the most important costimulatory interaction (51). Finally, activated liver B cells release TNFα and IL-6 (40), further stimulating T cell activation and polarization.

As a result, intrahepatic B cells promote T-cell activation during MASH and stimulate the generation of effector memory (EM) CD4 and CD8 T cells, which promotes inflammation during MASLD (40, 52). Compared to HFHC wildtype mice, livers from HFHC µMT mice show reduced percentages of EM CD4 and CD8 T cells and IFN-γ^+^ CD4 T cells. Similar effect can be achieved by anti-CD20 antibody-mediated B cell-depletion (40). Interestingly, the capability to activate liver T cells is likely to be restricted to intrahepatic B cells but not systemic B cells, as shown in experiments with HFHC-fed µMT recipients reconstituted with B cells from either the liver or spleen of HFHC-fed wildtype donor mice. Compared with recipients of spleen B cells, recipients of liver B cells showed worsened response in glucose tolerance test and insulin tolerance test, and increased frequency of IFN-γ^+^ CD4 T cells in the liver (40). These results indicate that the pro-inflammatory phenotype of B cells results from a local activation and is not a systemic event (38, 40).

B cell-mediated Th1 activation of CD4 T cells also plays an important role in M1 activation of hepatic macrophages in MASH. CD4 T-cell depletion in MASH mice significantly lowers the hepatic mRNA expression of macrophage M1 markers (42).

### 4.3 Interaction with macrophages

Macrophages are key players in MASLD development and progression (4). Liver macrophages represent a heterogeneous cell population that can be divided into two main categories: tissue-resident Kupffer cells and monocyte-derived macrophages (MoMFs) (4, 14). Kupffer cells initiate the inflammatory response in the liver and recruit monocytes to the liver. The recruited monocytes differentiate into pro-inflammatory macrophages, contributing to MASLD progression and fibrosis development (4). The pro-fibrotic effects of B2 cells involve the activation of liver macrophages and HSCs (40, 53). In MASLD, macrophages can be activated by a number of factors, including endotoxins and bacterial components due to increased intestinal permeability, factors released by damaged or dying hepatocytes, and nutritional lipids and their metabolites (4). Macrophage activation by B cells involves IgA and Fc receptor signaling (see below).

Activated macrophages promote MASLD progression by releasing proinflammatory cytokines and recruiting further immune cells (4). TNF and IL-1β are key cytokines in MASLD, driving the development of steatosis, inflammation and fibrosis, and the majority TNF and IL-1β molecules in the liver are derived from activated macrophages (54). In addition, macrophages also activate HSCs and myofibroblasts by releasing IL-6 and TGF-β, and are an important source of oxidative stress in MASLD (55). Finally, both Kupffer cells and infiltrating macrophages make a major contribution to MASLD development by recruiting neutrophils, B and T cells (56, 57).

### 4.4 Interaction with hepatic stellate cells

The activation of quiescent HSCs is a key event in liver fibrogenesis (58). Activated HSCs differentiate into myofibroblasts and contribute to fibrosis by secreting large amounts of collagen and profibrotic factors (53, 59). Interestingly, there exists a positive feedback mechanism in B cell-HSC interaction. Activated B2 cells strongly induce HSC activation by secreting pro-inflammatory cytokines including TNF-α; which is known to have a key role in promoting fibrosis (60). B cells are strong producers of TNF-α (40), and TNFα activates HSC by stimulating TNF-α receptor 1 (61). In turn, activated HSCs support liver B cell survival and maturation to plasma cells as well as B cell cytokine production by secreting retinoid acid (53).

### 4.5 Cytokine production

It has been known since the 1980s that B cells can be secrete a wide variety of cytokines (62, 63). Cytokine production by B cells is context-dependent; different stimuli lead to the production of different cytokines, with some being pro-inflammatory and others with anti-inflammatory properties (62). In steatosis (38) and MASH (40), intrahepatic B cells secrete elevated levels of IL-6 and TNF-α, which promote the activation of CD4 T cells and their differentiation into Th1 cells. As mentioned above, B cell-derived TNF-α is also involved in HSC activation (40), and TNF-α activates HSC by stimulating TNF-α receptor 1 (61). In addition to stimulating other immune cells, B cells also directly promote disease progression by releasing pro-fibrotic factors such as TGF-β1 and TIMP2 (40). TIMP2 is known to promote liver fibrosis through inhibition of extracellular matrix degradation (64).

### 4.6 IgG antibodies

Oxidative stress and lipid peroxidation are common characteristics of MASLD and MASH. Lipid peroxidation leads to the production of oxidized phospholipids and reactive aldehydes, including malondialdehyde (MDA) and 4-hydroxynonenal (4-HNE). These molecules activate hepatic inflammation by acting as danger-associated molecular patterns. In addition, they also form antigenic adducts with cellular macromolecules known as oxidation-specific epitopes (also called oxidative stress-derived epitopes, OSEs) (13, 14, 65, 66). MASLD patients have reduced levels of protective IgM natural antibodies against OSEs (see section 7) (67). In addition, elevated titers of pathogenic anti-OSE IgG have been detected in adult MASLD patients and in children with MASH (68). Especially, Anti-MDA IgG antibodies are associated with MASLD/MASH progression (68). High titers of anti-OSE IgG are associated with the severity of lobular inflammation and the prevalence of intrahepatic B/T cell aggregates (68), and are independently associated with increased risk of hepatic decompensation and mortality in MASH patients (69). Anti-OSE IgG is also an independent risk factor for MASH progression to fibrosis (68). In animal experiments, the increase in titers of anti-OSE IgG is associated with the maturation of liver B2 cells to plasma cells (41, 42), whereas reducing lipid peroxidation by supplementation with N-acetylcysteine attenuates steatohepatitis progression in a rat model (70). Conversely, immunization of MASH mice with MDA-adducted proteins stimulates IgG production and enhances transaminase release, lobular inflammation and fibrosis (42). It is likely that the resulting anti-MDA IgG antibodies promote MASH progression by inducing antibody-mediated injury (42).

## 5 Intestinal IgA^+^ B cells

In a recent study (16), strikingly different results were obtained in JH^-/-^ and µMT mice. The findings demonstrate that intestine B-cell responses are sufficient to induce MASH in mice (16).

In wildtype mice, choline-deficient high-fat diet (CD-HFD) induced MASH phenotypes. In contrast, JH^-/-^ mice were protected from CD-HFD-induced MASH, with reduced liver steatosis, lower MASLD activity score, normalized ALT levels and decreased hepatic lipid accumulation (16). In addition, fewer CD3^+^ T cells, CD8^+^ T cells, activated T cells (CD8^+^ CD62L^-^), TNF (CD8^+^ TNF^+^)- and IFNγ (CD8^+^ IFNγ^+^)-producing CD8^+^ cells were found in JH^-/-^ CD-HFD livers than in wildtype mice (16). To corroborate the results from these B cell-deficient mice, the authors depleted B cells therapeutically by using an anti-CD20 monoclonal antibody (αCD20 mAb) in wildtype MASH mice. The results from both the genetical and the therapeutical approaches were very similar and indicate a key role of B cell-response in the development of MASH.

Further remarkable results were obtained in µMT mice. These mice selectively develop IgA^+^ B cells in the gastrointestinal tract, but lacking B cells in the circulation or other organs (16, 31). Strikingly, CD-HFD-induced MASH levels in µMT mice were comparable to that in wildtype mice. CD-HFD µMT mice showed similar MASLD activity score, serum ALT levels, and hepatic CD8 cells accumulation as wildtype CD-HFD mice.

JH^-/-^ mice are a complete B cell-knockout model. In contrast, µMT mice have IgA^+^ B cells (only) in the intestine (see section 3.1). In wildtype mice, CD-HFD feeding led to an increase in IgA levels in the small intestine. Interestingly, IgA (although at lower levels) were present in the small intestine of µMT CD-HFD but not JH^-/-^ CD-HFD mice. IgM and IgG2b were absent in both µMT and JH^-/-^ mice (16). Importantly, MASH in µMT mice could be reversed by αCD20 antibody treatment, indicating that the susceptibility of µMT mice to MASH development was attributable to IgA^+^ B cells in the intestine. Indeed, MASH diet-induced accumulation of IgA^+^ B cells in small intestines was reduced by αCD20 antibody treatment (16).

Mechanistic data reveal that intestine B cells contribute to MASH by inducing CD8^+^ T cell-hyperactivation. Upon MASH diet intervention, intestinal B cells cluster with CD8^+^ T cells by a direct, antigen-independent cell-cell interaction stabilized by the co-stimulatory molecules LFA-1 and ICAM1. Moreover, intestinal B and T cells can efficiently migrate from the intestine to the liver. Thus, intestinal B cells are key players in MASH development by promoting T cell activation and transmigration to the liver. Another mechanism of B cells in MASH involves the secretion of IgA (16).

Normally, B cells in the gut-associated lymphoid tissues (GALTs) can be activated by protein antigens derived from gut microbiota bacteria with the help of CD4 T cells, leading to the generation of plasmablasts and plasma cells. In addition, T cell-independent activation by carbohydrate/polysaccharide antigens is also possible (71, 72). The intestinal B cells in the µMT mice on CD-HFD, however, are not plasmablasts or plasma cells (16). Increased intestinal B cells were also observed in CD-HFD-treated germ-free mice (16), indicating that intestinal B cells promote MASH independent of gut bacterial antigens. Rather, the intestinal B cells were activated metabolically carrying enhanced expression of gene related to BCR signaling, B cell-activation and lipid metabolism (16).

## 6 IgA

IgA is unique among all antibodies because it is produced to be secreted on the mucosal surface (73). As much as several grams of IgA are released into the human gut lumen everyday (71, 72). In the intestine, B cells are activated in the lamina propria via T cell-dependent (stimulated by bacterial antigens) or -independent mechanisms leading to the generation of IgA-producing plasma cells (71, 72). In addition, maternal milk IgA and liver-derived IgA also contribute to gut IgA pool (72). Gut IgA plays a key role in regulating microbiota composition and controlling enteric infections (71–73). The serum level of IgA is higher than that of IgM but much lower than that of IgG (74).

Elevated serum IgA levels have been demonstrated in patients with MASLD (72, 75, 76), and have been identified as an independent predictor of advanced fibrosis (75). MASLD is also accompanied by an accumulation of liver-resident IgA-producing plasma cells (9, 76, 77). However, serum IgA levels do not correlate with MASLD severity in pediatric patients (78). It is still unclear whether this discrepancy is due to the difference in the microbiota composition between children and adults or other reasons.

A recent study has provided evidence showing that IgA can promote the initiation of liver fibrosis by driving the activation of MoMFs in the liver (16).

CD-HFD-induced liver fibrosis is absent in IgA^-/-^ mice lacking secreted IgA (79) or in wildtype mice treated with αCD20 antibody, indicating that the presence of B cells and an intact IgA secretion in the periphery are obligatory for fibrosis development. Moreover, the degree of liver fibrosis correlates with serological IgA levels in several diet-induced MASH models (16).

Macrophages play a crucial role in liver injury and fibrosis in MASH, with MoMFs exhibiting a proinflammatory phenotype that can directly activate HSCs (15). IgA drives MoMF activation via Fc receptor signaling. Diet-induced MASH in wildtype mice is associated with an increased level of MoMFs, and MoMFs are associated in number with the degree of fibrosis. Interestingly, liver MoMFs, but not other immune cell populations, have strongly upregulated FcR genes and genes involved in FcR signaling activation in all tested MASH models (16). Conversely, FcRγ^-/-^ mice on CD-HFD diet develop steatosis, liver damage, and inflammation comparable to wildtype mice, yet liver fibrosis is markedly reduced in these animals (16). *Ex vivo* experiments demonstrate that serum IgA from MASH mice triggers macrophage activation and induction of profibrogenic genes (IL-1β, IL-6 and TNF) in a manner dependent on Fc receptor signaling (16).

Notably, Fc receptors are also expressed on other immune cells including NK cells (71). Therefore, future studies should investigate whether Fc-mediated mechanisms by IgA also applies to other immune cells.

## 7 B1 cells and IgM

In addition to B2 cells, a small fraction of B1 cells is also found in the mouse liver (40, 80). These cells are predominantly B1b cells (CD3^−^ NK1.1^−^ CD19^+^ CD23^−^ CD5^−^ B220^lo^ IgM^+^ IgD^−^), whereas CD5^+^ B1a cells are almost undetectable (40). Upon HFHC feeding, the number of B1b cells is reduced in the MASH mice (40).

B1 cells are named as such because they are derived from the fetal origins and their development is earlier than that of the B2 cells in the bone marrow (81). B1 cells represent the majority of B cell populations in the peritoneal and pleural cavities of mice and their number is maintained by self-renewal. If activated by LPS, IL-5 or IL-10, B1 cells migrate from the body cavities to the spleen or lymph nodes, starting to secrete cytokines or natural IgM antibodies without the need of foreign antigen exposure (81).

Although B1 cells differentiate “spontaneously” into natural antibody-producing cells without requiring foreign antigens or CD4 T cell help, immunization with antigens can lead to dynamic expansion of B1 cell repertoire through BCR-mediated mechanisms (81). The immunization of mice with heat-inactivated pneumococci results in the enhanced production of natural IgM antibodies against bacterial antigens that cross-react with oxidized phosphatidylcholine in LDL molecules (82). The engulfment of oxidized LDL by Kupffer cells is known to promote Kupffer cell activation and hepatic inflammation. Thus, the interaction of IgM with oxidized LDL results in a reduction in their uptake by Kupffer cells, thereby lowering the pro-inflammatory stimuli (83). This may explain why the immunization-induced production of natural IgM leads to a reduction of MASH in LDL receptor-deficient mice receiving a high-fat/cholesterol diet (82).

In patients with MASLD, IgM antibody titers towards different OSE (including MDA and malondialdehydeacetaldehyde) are lower than in healthy control individuals (67). Moreover, the data suggest that obesity/hyperlipidemia is a causal factor for the reduced IgM levels (67). Nevertheless, more studies are needed to decipher the roles of B1 cells and IgM antibodies in MASLD and MASH (65).

## 8 Regulatory B cells

Notably, the same mouse line can exhibit quite different phenotypes when put on different MASLD- or MASH-inducing diets (**Table 1**). For example, the MASH phenotype of µMT mice fed CD-HFD is comparable with wildtype mice (16). On HFHC diet, in contrast, µMT mice develop markedly less MASH than wildtype mice (40). Similar situation has been observed for the IgMi mice. In the MASH model induced by CD-HFD, IgMi mice are equally protected from MASH as JH^-/-^ mice (16). In the HFD-induced obesity model, however, we have observed significant differences between IgMi and JHT mice (36).

As detailed in section 3, JHT and JH^-/-^ mice are completely devoid of mature B cells, whereas IgMi mice represent a model of antibody-deficiency. The IgMi mice have normal number of B cells (36, 37), producing cytokines but not secreted antibodies (28, 35). Moreover, the adaptive immune responses remain largely intact (16).

In a recent publication, we put wildtype mice, JHT and IgMi mice on HFD for 21 weeks (36). In wildtype mice, HFD feeding resulted in MASLD with massive macrovesicular steatosis, modest hepatic and adipose tissue inflammation, and incipient liver fibrosis. JHT mice showed reduced HFD-induced MASLD, confirming the pathogenic role of B cells in MASLD (36). Strikingly, the IgMi mice were completely protected from the development of hepatic steatosis, inflammation, and fibrosis. Our study indicates that B cells may also have protective roles, in addition to the pathogenic effects in MASLD.

The common feature of JHT and IgMi mice is that they do not secrete antibodies, whereas HFD feeding in wildtype mice led to increased levels of serum IgG2c (36). The lack of pathogenic antibodies (IgA and IgG) should protect JHT and IgMi mice to a similar extent. Whereas JHT mice have no mature B cells at all, the number of B cells in the IgMi mice remains normal. The fact that the IgMi mice are more protected than the JHT mice indicates that the additional protection observed in the IgMi mice may be attributable to the presence of certain B cell subsets. The results from our study indicate that regulatory B cells (Breg) are likely a candidate subset for these protective B cell populations.

Breg cells represent a minor B cell population with immunomodulatory functions by producing IL-10, IL-35 and TGF-β (51, 84). Although the existence of Breg cells is beyond doubt, it remains difficult to define and identify Breg populations (51). In addition to the IL-10-producing CD1d^high^CD5^+^ cells, various other Breg populations have been reported (23, 51). Until now, there is no unified transcription factor or lineage-specific marker defined for Breg cells, and the identification of Breg cells by surface markers has been controversial (23, 51). Production of IL-10 is considered the major characteristics for the discrimination of Breg from conventional B populations (51).

In our study, CD1d^high^CD5^+^CD19^+^B220^+^ Breg cells were found in the liver of both wildtype and IgMi mice. HFD feeding reduced Breg cell number and IL-10 production in the liver of wildtype mice, whereas Breg cell number and IL-10 expression were increased in HFD-fed IgMi mice. Among the CD1d^high^CD5^+^ Breg cells, the number of CD5^+^ IL-10^high^ cells almost doubled in IgMi mice on HFD. Moreover, livers of patients with advanced liver fibrosis showed abundant deposition of IgG and stromal B cells and low numbers of IL-10 expressing cells, supporting our mouse data (36).

These results suggest that B lymphocytes have both detrimental and protective effects in HFD-induced MASLD. IL-10-producing regulatory B cells may represent such a protective B cell subset. Nevertheless, evidence for a causal role of Breg cells is still missing (36). The cytokine IL-10 (62, 85, 86) is a key anti-inflammatory mediator produced not only by Breg cells, but also by various cell types, including B1 and B effector (Be) Be2 cells (62, 81). Therefore, more experimental evidence is needed to verify the identity of the protective B cell subset, and the cellular source of IL-10 in the MASLD liver.

## 9 Conclusion and future directions

Accumulating evidence supports the pathogenic role of B cells in MASLD and MASH. B2 cells can be activated by oxidation-specific epitopes, by gut microbiota metabolites or bacterial protein antigens. Activated B2 cells promote MASLD and MASH by activating T cells, macrophages, hepatic stellate cells, by producing pathogenic IgG and IgA antibodies, and by releasing pro-inflammatory, pro-fibrotic factors. In addition, there is emerging evidence for a protective role of certain B cell subsets in MASLD and MASH. Nevertheless, more studies are warranted to verify the identity of these cells and the detailed mechanisms involved. Future studies should explore the therapeutic potential of approaches targeting B2 cells or the downstream mechanisms. Conversely, approaches that enhance the protective effects of Breg cells, B1 cells and natural IgM antibodies may also be promising.
